# TAILORED ACTIVITY PROGRAM (TAP) COSTS: OPPORTUNITIES TO STREAMLINE DELIVERY AND PURSUE REIMBURSEMENT

**DOI:** 10.1093/geroni/igz038.651

**Published:** 2019-11-08

**Authors:** Laura T Pizzi, Laura T Pizzi, Katherine M Prioli, Eric Jutkowitz, Jing Yuan, Laura N Gitlin

**Affiliations:** 1 Rutgers University, Piscataway, New Jersey, United States; 2 Brown University, Providence, Rhode Island, United States; 3 Drexel University, Philadelphia, Pennsylvania, United States

## Abstract

TAP intervention costs captured alongside the randomized controlled study included labor of program staff, mileage, supplies and materials. Staff time costs were converted to $US 2017 by multiplying hours spent by the appropriate wage rate plus fringe benefits; mileage was costed using the federal reimbursement rate. Research costs were excluded to approximate real world implementation. Costs varied based on number of visits required but mean component costs per dyad were: occupational therapist training ($133.50), home visit time ($527.57), travel ($718.02), work outside of intervention delivery ($57.14), program screening for eligibility ($3.73), supervision for quality assurance ($250.23), activity supplies ($51.64), and program materials ($29.32). Findings will be compared to a 2008 TAP pilot study post hoc cost analysis to identify how broader scale implementation impacts intervention costs. Opportunities to streamline program delivery will be discussed as well as potential reimbursement mechanisms via Medicare part B, Medicare Advantage, and Medicaid waiver programs.

